# Hepatitis C Virus Infection as a Traumatic Experience

**DOI:** 10.1371/journal.pone.0110529

**Published:** 2014-10-23

**Authors:** Mychelle Morais-de-Jesus, Renato Daltro-Oliveira, Karine Miranda Pettersen, Adriana Dantas-Duarte, Luciana Di-Domizio Amaral, Patrícia Cavalcanti-Ribeiro, Carlos Teles Santos, Maria Isabel Schinoni, Liana R. Netto, Lucas Araújo-de-Freitas, Raymundo Paraná, Ângela Miranda-Scippa, Karestan C. Koenen, Lucas C. Quarantini

**Affiliations:** 1 Programa de Pós-graduação em Medicina e Saúde, Faculdade de Medicina da Universidade Federal da Bahia, Salvador, BA, Brazil; 2 Faculdade de Medicina da Universidade Federal da Bahia, Salvador, BA, Brazil; 3 Pontifícia Universidade Católica de São Paulo, São Paulo, SP, Brazil; 4 Departamento de Ciências Exatas, Universidade Estadual de Feira de Santana, Feira de Santana, BA, Brazil; 5 Instituto de Saúde Coletiva, Universidade Federal da Bahia, Salvador, BA, Brazil; 6 Hospital Universitário e Departamento de Neurociências e Saúde Mental da Faculdade de Medicina da Bahia, Universidade Federal da Bahia, Salvador, BA, Brazil; 7 Department of Epidemiology, Mailman School of Public Health - Columbia University, New York, New York, United States of America; Harvard Medical School, United States of America

## Abstract

**Objective:**

The purpose of this study was to evaluate whether individuals consider their HCV infection to be a potentially traumatic experience. Additionally, we investigated its association with Post-Traumatic Stress Disorder (PTSD) and the impact of PTSD diagnosis on health-related quality of life (HRQoL) in HCV infected subjects.

**Methods:**

We conducted a cross-sectional survey of 127 HCV-infected outpatients recruited at a University Hospital in Salvador, Brazil. All subjects answered an orally-administered questionnaire to gather clinical and socio-demographic data. We investigated traumatic experiences and the subject's perception of the disease using the Trauma History Questionnaire. PTSD and other psychiatric diagnoses were assessed through the Mini International Neuropsychiatric Interview-Brazilian Version 5.0.0 (M.I.N.I. PLUS). HRQoL was assessed using Short-Form 36 (SF-36).

**Results:**

Approximately 38.6% of the patients considered hepatitis C to be a traumatic experience. Of these, 60.7% had a PTSD diagnosis. PTSD was associated with significant impairment in quality of life for individuals in seven SF-36 domains as shown bymultivariate analysis: Role-Physical (β: −24.85; 95% CI: −42.08; −7.61), Bodily Pain (β: −19.36; 95% CI: −31.28; −7.45), General Health (β: −20.79; 95% CI: −29.65; −11.92), Vitality (β: −11.92; 95% CI: −20.74; −3.1), Social Functioning (β: −34.73; 95% CI: −46.79; −22.68), Role-Emotional (β: −26.07; 95% CI: −44.61; −7.53), Mental Health (β: −17.46; 95% CI: −24.38; −10.54).

**Conclusion:**

HCV is frequently a traumatic experience and it is strongly associated with PTSD diagnosis. PTSD significantly impaired HRQoL.

## Introduction

Infection with hepatitis C virus (HCV) is common throughout the world and can result in severe liver damage and failure. Worldwide, the most common route of HCV transmission is intravenous drug use. The main risk factors in Brazil are blood transfusions from non-tested blood donors, intravenous drug use, and invasive therapies with contaminated equipment [1]. Additional risk factors of HCV infection include perinatal infection, sexual transmission and activities with potential exposure to blood, such as tattoo and piercing. The disease can manifest in a wide spectrum of effects, ranging from various degrees of liver damage, syndromes of immunological origin — such as rheumatoid symptoms and cryoglobulinemia [2]–[4] — and neuropsychiatric disorders such as depression and cognitive deficits [5]–[12].

Psychiatric disorders are highly prevalent among HCV-infected patients, and have a well established effect on their well-being. PTSD is a common, debilitating, complex syndrome which occurs in some persons after personal exposure to a traumatic stressor or experienced by a family member or another close associate. Some of the main symptoms are intense fear, helplessness, persistent avoidance of stimulus associated with the trauma, increased arousal. [13]. “The full symptom picture must be present for over 1 month (Criterion E), and the disturbance must cause clinically significant distress or impairment in social, occupational, or other important areas of functioning” (DSM-IV). Populational studies show the disorder's prevalence to be around 6.8% in a study carried out in the United States [14] and 14% in another one carried out in Brazil [15]. PTSD is highly comorbid with many different medical and psychiatric illnesses and can be underestimated in non-clinical populations [15]–[17]. It is also associated with significantly low rates of HRQoL [18], [19]. PTSD has already been cited in HCV populations in a small number of studies, most of which were primarily focused on other specific populations, such as veterans, HIV co-infected persons, and persons on dialysis [20]–[25].

Patients carrying the virus usually have a lower health-related quality of life (HRQoL) when compared with a reference population [26]–[28], even in the absence of severe hepatic disease [29], [30]. HRQoL is a multidimensional concept embracing an individual's perception of their own well-being in terms of: physical functioning; role-physical; bodily pain; general health; vitality; social functioning; role-emotional; and mental. This concept is based on the World Health Organization's definition of health as “A state of complete physical, mental, and social well-being and not merely the absence of disease or infirmity” [31].

The cause of the reduced HRQoL in asymptomatic HCV-infected patients is not well understood and may have a multifactorial origin. Fatigue and other symptoms of a neuropsychiatric nature, like forgetfulness, depression and difficulty concentrating, are common complaints of HCV-infected patients [5], [29]. These patients also perform more poorly on neuropsychiatric tests [5], [32], [33], suggesting the virus may act on the nervous system. Evidence of HCV replication in the central nervous system [34], [35] supports this hypothesis. HRQoL is also improved in patients who achieve sustained virological response after antiviral therapy [36]–[38].

Another line of research considers that personal background may affect HRQoL due to low household income, intravenous drug use, coexistent co-morbidities, knowledge of diagnosis, and other factors associated with acquiring the infection [39], [40].

Patients with a comorbid psychiatric disorder and HCV-infection are well documented to have reduced HRQoL [41], [42]. The impact of depression on HRQoL in these patients is well-investigated [43], [44]. However, the relationship between post-traumatic stress disorder (PTSD) and HCV infection, and their combined consequence on HRQoL, is not well studied.

Accordingly, this study aimed to evaluate whether HCV-infected individuals consider their HCV infection to be a potentially traumatic event, and its association with PTSD diagnoses. The secondary objective is to investigate the relationship between PTSD diagnosis and HRQoL in HCV-infected patients. We hypothesized that, among hepatitis C patients, there is a perception of hepatitis C disease as a potentially traumatic experience, as well as an association between PTSD diagnosis and poorer HRQoL.

## Materials and Methods

### Ethics statement

Ethical approval for this study was obtained from the local MCO – UFBA, Institutional Review Board (IRB) (protocol 14/2002) and it is in accordance with the Helsinki Declaration of 1975. Prior to any data collection and after a complete description of the research, written informed consent was obtained from the patients who agreed to participate. All subjects answered an orally-administered questionnaire to gather clinical and socio-demographic data.

### Subjects and data collection

We conducted a cross-sectional survey with chronic hepatitis C outpatients from the University Hospital (Com-HUPES) – Federal University of Bahia (UFBA) between June 2009 and July 2012. We included all patients older than 18 years of age in consecutive order. A total of 127 patients diagnosed with chronic hepatitis C were enrolled in the study (men  = 82, women  = 45). The samples were divided into ninety-nine HCV-control subjects (78%) and twenty-eight HCV-PTSD subjects (22%).

### Measurement Instruments

We used the Trauma History Questionnaire (THQ), a 24-item self-report measurement, to assess subjects' history of traumatic life events. The adaptation of the scale for Portuguese met the criteria of semantic and operational equivalences [45]. The THQ is suitable for clinical and research environments. Traumatic experiences were classified into the following categories: “crime-related events”, “general disaster and trauma”, “physical and sexual experiences”, and “other events”.

The subjects were also asked about the frequency of the event and their ages when it occurred.

In addition to the analysis of these four types of traumatic experience, subjects who answered “Yes” to question fifteen were analyzed separately: “Have you ever had a serious or life-threatening illness? If yes, please specify.” This question investigated whether the patient considered hepatitis C to be a stressful, life-threatening experience that was experienced as a potentially traumatic event, and may be associated with PTSD.

Axis I clinical syndromes were diagnosed using the Mini International Neuropsychiatric Interview-Brazilian Version 5.0.0 (M.I.N.I. PLUS), a short, structured diagnostic interview compatible with DSM-IV and ICD-10 criteria [46].

Quality of life was assessed using Short-Form 36 (SF-36) [47]. SF-36 has been used for numerous studies all over the world in order to assess quality of life, and has been translated and validated in Brazil. The scale was translated and made suitable for the socio-economic and cultural Brazilian conditions, where validity and reproducibility were demonstrated [48]. The HRQoL was measured by eight domains: physical functioning; role-physical; bodily pain; general health; vitality; social functioning; role-emotional; and mental health. The final scores in each domain were adjusted linearly from 0 to 100 via the formulas found in SF-36; a score of “0” indicates the worst health status, a score of “100” indicates the most favorable health status.

All interviews were conducted face-to-face by trained mental health professionals. Kappa index (proportion of agreement beyond probability divided by the agreement potential) was used to obtain reliability. Every patients' disorder was recorded and fulfilled the criteria, according to this tool. The Kappa index analysis among observers in the M.I.N.I. was 0.9.

### Data Analysis

Statistical analysis was conducted with STATA 9.0 software. The subjects were divided into two groups according to diagnosis for post-traumatic stress disorder: HCV without PTSD symptoms (HCV-control) and HCV with a diagnosis of PTSD (HCV-PTSD). The following independent variables were selected with the objective of performing descriptive analyses between these groups: gender, age, civil state, occupation, transplant eligibility, presence of medical comorbidity, number of medical comorbidities, presence of psychiatric comorbidity and number of psychiatric comorbidities. Chi-Square and Kruskall-Wallis tests were used to compare categorical and continuous variables, respectively, between HCV-control and HCV-PTSD groups.

To evaluate whether any THQ categories or a positive response to question fifteen of THQ were independently associated with the severity of disease (determined by transplant eligibility) or a diagnosis of PTSD, the Pearson test was used.

Bivariate analyses were carried out to verify the possible differences between PTSD diagnosis and HRQoL scores through the non-parametric Pearson test. In order to control for potential confounding factors, a multivariate analysis was performed by a robust linear regression model that allowed us to estimate the increment (or decrement) yield (β) when patients with PTSD were compared with the reference group, patients with a negative PTSD diagnosis.

All tests were performed using the significance level of 5% (p≤0.05).

## Results

The HCV-control group was predominantly male (68.7%), ranging in age from 23 to 68 years (mean  = 53±8.8); most subjects had a stable partner (64.65%); over 69% had a paid occupation; 49% were eligible for a liver transplant; 44.1% had some decompensated or systemic medical comorbidity; and 50% had a psychiatric comorbidity (Table 1). On the other hand, the HCV-PTSD group had a balanced gender variable (14 male and 14 female), and ranged in age from 28 to 65 years (mean  = 52±9.27); 53.57% were in a civil partnership; most had a paid occupation (75%); 78.57% were not eligible for a transplant; 42.86% had a decompensated or systemic medical comorbidity; and 71.43% had another psychiatric comorbidity. Significant differences between groups were found through bivariate analysis on the variables: civil state (p = 0.03) and number of psychiatric comorbidities (p = 0.002) (Table 1).

**Table 1:** Socio-demographic and clinical characteristics.

Variables	HCV-control	HCV-PTSD	
	(99 subjects)	(28 subjects)	*p* value
**Gender**			
Male N (%)	68 (68.70)	14 (50.00)	
Female N (%)	31 (31.31)	14 (50.00)	0.06*
**Age** Mean (SD)	53 (8.80)	52 (9.27)	0.57**
**Civil state**			
Single N (%)	21 (21.21)	5 (17.86)	
With stable partner N (%)	64 (64.65)	15 (53.57)	
Divorced N (%)	12 (12.12)	4 (14.29)	0.03*
Widower N (%)	1 (1.01)	4 (14.29)	
**Paid occupation**			
Without N (%)	16 (16.67)	5 (17.86)	
With N (%)	67 (69.79)	21 (75.00)	
Retired N (%)	13 (13.54)	2 (7.14)	0.66*
**Transplant eligibility**			
No N (%)	50 (50.10)	22 (78.57)	0.08*
Yes N (%)	49 (49.90)	6 (21.43)	
**Medical comorbidity**			
No N (%)	47 (47.47)	16 (57.14)	0.36*
Yes N (%)	52 (52.53)	12 (42.86)	
**Number of medical comorbidities** Mean (SD)	0.65 (0.74)	0.75 (1.04)	0.84**
**Psychiatric comorbidity**			
No N (%)	48 (48.48)	8 (28.57)	0.06*
Yes N (%)	51 (51.52)	20 (71.43)	
**Number of Psychiatric Comorbidities** Mean (SD)	0.79 (1.02)	1.68 (1.56)	0.00**

SD  =  Standard Deviation;

* *χ^2^* Test;

** Kruskall-Wallis Test.

The experiences of: “crime-related events,” “general disaster and trauma,” and “other events” showed no significant association with the development of PTSD diagnosis, unlike “physical and sexual experiences,” which showed a significant association (p = 0.019). Of all subjects, 38.6% considered hepatitis C to be a traumatic experience. Of these, 60.7% had a PTSD diagnosis. The perception of hepatitis C as a traumatic experience was not associated with disease severity (needing a transplant), however it showed a significant association with diagnosis of PTSD (p = 0.003).

Bivariate analysis revealed a negative impact of PTSD symptoms in the following SF-36 domains: role-physical, bodily pain, general health, vitality, social functioning, role-emotional and mental health. Multivariate analysis reported a negative association in the following domains: role-physical, bodily pain, general health, vitality, social functioning, role-emotional and mental health, that remained significant even after adjusting for confounding factors (Table 2).

**Table 2:** Bivariate and Multivariate Analysis of PTSD diagnosis and Quality of Life in Chronic Hepatitis C Patients.

SF-36 Domains	HCV-control (99)	HCV-PTSD (28)	Bivaried Analysis	Multivaried Models*
	Mean (SD)	Mean (SD)	β* [CI95%]	β* [CI95%]
**Physicalfunctioning** **	75.00 (24.32)	68.00 (27.01)	−6.78 [−17.76; 4.19]	−6.15 [−16.42; 3.11]
**Role-physical** **	59.94 (41.20)	36.6 (42.65)	−23.34 [−40.59; −6.08]	−24.85 [−42.08; −7.61]
**Bodily pain** **	73.56 (25.49)	51.5 (26.6)	−22.06[−33; −11.11]	−19.36 [−31.28; −7.45]
**General health** **	65.20 (19.26)	46.03 (23.39)	−19.16[−28.52; −9.81]	−20.79 [−29.65; −11.92]
**Vitality** **	68.41 (22.45)	49.64 (25.45)	−18.77[−29.07; −8.47]	−11.92 [−20.74; −3.1]
**Social functioning** **	78.94 (25.29)	42.85 (27.09)	−36.08[−47.17; −24.99]	−34.73[−46.79; −22.68]
**Role-emotional** **	82.63 (31.48)	46.42 ( 43.82)	−36.21[−53.38; −19.03]	−26.07[−44.61; −7.53]
**Mental health** **	81.14 (15.33)	56.71 (21.77)	−24.42[−32.93; −15.91]	−17.46[−24.38; −10.54]

*Standardized-β coefficients (Betas);

**Adjustment: gender, transplant eligibility, anxiety disorders, other psychiatric comorbidities, current depressive episode, past depressive episode, number of psychiatric comorbidities.

## Discussion

For our knowledge, this is the first study demonstrating HCV as a traumatic experience strongly associated with PTSD. The prevalence of PTSD in this sample was 28%, which is extremely high compared to mean prevalence in the general world population, ranging from 0% to 10.4% [49]. A recent survey carried out in Brazil, in the two largest cities in the country, São Paulo and Rio de Janeiro, has estimated the prevalence of PTSD in one year to range between 1.2% and 7.8% [50].

Our finding that a high proportion of HCV patients considered the condition to be a potentially traumatic experience is consistent with previous studies investigating severe acute respiratory syndrome (SARS) [51] and myocardial infarction [52], which are both recognized as potentially traumatic events that may lead to post-traumatic stress syndrome. However, one limitation of the survey is that the trauma can be influenced by the fear of death or stress of living with an illness, since the question “Have you ever had a serious or life-threatening illness?” can be interpreted differently by each patient.

The disease severity was not significantly associated with either the perception of hepatitis C as a traumatic experience or PTSD diagnosis. Contrary to our expectations, disease severity showed a tendency of negative association with PSTD diagnosis. This unexpected result is possibly related to the small sample size, and requires further investigation.

Even after adjusting for psychiatric comorbidities, PTSD was associated with quality of life scores significantly lower in seven of the eight SF-36 domains, as shown by bivariate and multivariate analysis. Similar results were found in other studies that analyzed quality of life in individuals diagnosed with PTSD symptoms [53], [54], including individuals with a past diagnosis of non-recurring PTSD [55]. Therefore, PTSD is related to impaired mental and physical health as well as increased healthcare utilization [56].

Consistent with other studies [57], there was a higher prevalence of PTSD diagnosis in female subjects (45.16%) compared to males (20.58%). The prevalence of psychiatric disorders in both HCV-control and HCV-PTSD groups was high, especially the latter, which is in accordance with previous clinical studies regarding individuals infected with hepatitis C [10], [43]. The HCV-PTSD samples had a prevalence of psychiatric disorders 49% higher on average than the general Brazilian population, according to estimates from epidemiological studies of the general population [58]. Although the majority of the studies relating PTSD to HCV patients has been conducted with veterans and HIV co-infected patients, this study had only two HIV co-infected patients and no veterans.

The mode of HCV contraction is potentially an important variable to explain some of the trauma; therefore it is important to highlight that a previous study in the population of Bahia, Brazil, found that the most common route of HCV infection is through unscreened blood transfusions [42]. Furthermore, none of the patients cited it as a traumatic experience when they were asked about the history of potentially traumatic experiences through the Trauma History Questionnaire.

The findings of this study should be interpreted with consideration of the following methodological limitations: the cross-sectional design may cause bias for assessment of other psychiatric correlations with PTSD. Since stigmatization is an important phenomenon in the HCV-infected population [59]–[61], patients may, as an avoidance behavior, under-report their psychopathology. The study design also made it impossible to know whether the perception of HCV as a traumatic experience was impacted by pre-existing PTSD, or was the source of the PTSD itself. Finally, the small sample size of this study may limit the power to detect association factors with moderate strength.

This study contributes to HCV being perceived as a traumatic experience and, furthermore, reveals a high prevalence of PTSD among HCV-infected patients. Our findings show that considering a subject's perception of HCV as a traumatic experience may help to detect PTSD; a condition with significant HRQoL impairment.
